# Photonics with Gallium Nitride Nanowires

**DOI:** 10.3390/ma15134449

**Published:** 2022-06-24

**Authors:** Norah Alwadai, Nigza Saleman, Zainab Mufarreh Elqahtani, Salah Ud-Din Khan, Abdul Majid

**Affiliations:** 1 Department of Physics, College of Sciences, Princess Nourah bint Abdulrahman University, P.O. Box 84428, Riyadh 11671, Saudi Arabia; nmalwadai@pnu.edu.sa (N.A.); zmelqahtani@pnu.edu.sa (Z.M.E.); 2 Department of Physics, Hafiz Hayat Campus, University of Gujrat, Gujrat 50700, Pakistan; nigza.saleman@yahoo.com (N.S.); abdulmajid40@yahoo.com (A.M.); 3 Sustainable Energy Technologies Center, College of Engineering, King Saud University, P.O. Box 800, Riyadh 11421, Saudi Arabia

**Keywords:** plasmons, GaN, coupling, nanowires, final element analysis

## Abstract

The surface plasmon resonance in low-dimensional semiconducting materials is a source of valuable scientific phenomenon which opens widespread prospects for novel applications. A systematic study to shed light on the propagation of plasmons at the interface of GaN nanowire is reported. A comprehensive analysis of the interaction of light with GaN nanowires and the propagation of plasmons is carried out to uncover further potentials of the material. The results obtained on the basis of calculations designate the interaction of light with nanowires, which produced plasmons at the interface that propagate along the designed geometry starting from the center of the nanowire towards its periphery, having more flux density at the center of the nanowire. The wavelength of light does not affect the propagation of plasmons but the flux density of plasmons appeared to increase with the wavelength. Similarly, an increment in the flux density of plasmons occurs even in the case of coupled and uncoupled nanowires with wavelength, but more increment occurs in the case of coupling. Further, it was found that an increase in the number of nanowires increases the flux density of plasmons at all wavelengths irrespective of uniformity in the propagation of plasmons. The findings point to the possibility of tuning the plasmonics by using a suitable number of coupled nanowires in assembly.

## 1. Introduction

Nanomaterials have revealed very interesting and novel phenomena that have never been observed in bulk materials. The interaction of light with nanomaterials introduces collective oscillations of electrons known as surface plasmons, which can be exploited in a variety of devices and applications. The study of surface plasmons is important to understand material properties, due to which it emerged as an interdisciplinary research field referred to as plasmonics [1]. It provides a route to sub-wavelength optics which provides access to several novel phenomena and has potential for a number of applications in optoelectronic and photovoltaic devices [2]. Initially, metals were used at the nanoscale to exploit plasmonic properties. However, with improvements in synthesis techniques, the relevant prospects of compound semiconductors have been investigated [3].

Plasmon-based photonics helps investigate the mechanism of light–matter interactions at sub-wavelength scales, which provides a basis for novel applications [4]. The nano-photonics technology based on plasmonics helps the realization of optoelectronic devices to generate, modulate and detect light. There have been extensive research efforts to utilize metallic nanomaterials for plasmonic applications [5]. However, considering the limited modification capability of metals, the application of tunable semiconductors in plasmonic devices have been tested as alternate materials. The heavily doped semiconductors exhibiting metallic-like characteristics with Fermi level shifted inside the principal bands have shown surface plasmon resonance (SPR). The phenomenon of SPR in semiconductors in the IR and mid-IR region has been found to be useful for plasmonic-related novel applications. The SPR in the mid-IR region has been observed in ZnO thin films using ellipsometry and theoretical predictions of reflectivity simulations [6]. The segregation appeared to strongly affect SPR and the negligible role of ionized impurities on plasmonic damping. The quantum dots (QDs) of wide band gap ZnO have shown infrared SPR which can be controlled via surface redox reactions [7]. Transparent ZnO has also been utilized to detect plasmonic features in metal–insulator–metal layered nanostructures [8]. The metallic part of the structure was composed of layers of heavily Ga doped ZnO on two ends, whereas undoped ZnO as the insulating dielectric was sandwiched in between. The gap SPR could be tuned on the basis of variation in the thickness of the dielectric and the layers. The anisotropy in CuS nanocrystals exhibited coherent excitations per analysis of experimental extinction spectra and theoretical predictions [9]. In an attempt to study dopant distribution in semiconductors, the segregation of tin in the surface of indium tin oxide has shown an influence on dopant activation in the matrix [10]. The role of rich Cu vacancies and hence valance band holes on SPR in CuP nanocrystals studied on the basis of scanning transmission electron microscopy measurements has been reported [11]. NMR spectroscopy of copper solenoid with different Cu compositions exhibited plasmonic characteristics in a low doped regime that depends on the charge density of the carriers [12].

Gallium nitride (GaN), owing to its exceptional chemical and physical properties, is a potential candidate for plasmonic applications [13]. The nanowires of GaN have been found robust to be for electronic and optoelectronic devices at the nanoscale [14]. In an earlier effort, phonon–plasmon coupling has been recorded using Raman spectroscopy in GaN films with different electronic concentrations [15]. GaN-based metal-semiconductor layers have been found to be useful for plasmonic applications in telecommunications [16]. The study points to the potential of GaN for exploiting in semiconductor-based structures to prepare modulators and switches. Recently, a GaN-based heterostructure with WS_2_ has shown promise for a sensitive SPR sensor working at operational wavelengths [17]. The role of SPR in the performance of GaN LEDs has been examined by performing detailed spectroscopic measurements [18]. Colloidal Ag nanoparticles were coated on the surface of p-GaN via chemical etching to produce a rough surface that exhibited improved quantum efficiency. The deposition of metallic particles on GaN nanostructures has also been performed to study prospects of SPR applications. Gold nanoparticles were decorated on GaN nanostructures to prepare UV photodetectors [19]. The quantum efficiency and photo-response of the device were found to be significantly enhanced due to the Au nanoparticles. The study helped explore the interaction of light with SPR-guided nanoparticles, shedding light on the production of efficient optoelectronic devices for future applications.

The application of nanowires in optical devices has shown excellent performance due to high index contrast, high sensitivity, minimal losses and small voltage requirements. So far, the majority of research efforts to utilize SPR in nanowires have been dedicated to metals. The nanowires have been used as optical sensors because they have the advantage of high index contrast and low optical power losses. In contrast to other nanostructures, nanowires are able to produce polarized light, which finds dedicated applications in photonics. It has been predicted that SPR in nanowires travels in a helical order down the wire in response to polarization of the input radiations [20]. The plasmons, while travelling down the nanowires, are reflected from the end, which exhibits a cavity-like property of the extended wires. This character of the nanowires was observed when a reduction in the group velocity of the plasmons was noted in Ag nanowires of different diameters [21]. Plasmon damping, which may help design waveguide structures, also takes place due to the bending of nanowires and substrate effects. The metallic nanowires can be used as sources of switchable surface plasmons. Bharadwaj et al. investigated electronic tunneling between Au nanowires and the Au tip in a scanning tunneling microscope to explore the excitation of plasmons [22]. The coupling of plasmons in the Ag nanowires was detected at the exit and the results pointed to the generation of switchable plasmons. It has recently been reported that semiconducting nanowires can be tuned to obtain surface-enhanced Raman scattering [23]. Graphene doped TiO_2_ has been found to offer excellent photocatalytic properties in the visible part of spectrum [24].

The further potentials of plasmonic nanowires include the coupling of the nanowire with emitters, SPP routing, SPP interference, controlled optical designs, interfacing, and information processing, which is not only helpful for preparing future functional nanophotonic devices but also for exploring the fundamental mechanisms involved [25]. However, despite the availability of basic knowledge on plasmonics in metal nanowires, much less in known on semiconducting nanowires. This work is dedicated to a comprehensive study to explore plasmon formation in coupled and uncoupled GaN nanowires. GaN is a well-known material for its electronic and optoelectronic applications [26,27]. The hybrid semiconducting structures comprising GaN exhibits excellent device grade optical properties [28]. It is the first work on GaN nanowires to shed light on propagation of the plasmons along the designed geometries comprising single, double and four nanowires. The coupled and uncoupled plasmons in the structures are investigated and the effect of an increase in the number of nanowires on the flux density of plasmons is studied.

## 2. Method

The calculations were based on the finite element method (FEM) and carried out using the Semiconductor and Coefficient Form PDE module of COMSOL Multiphysics [29]. The FEM is used to solve partial differential equations by using finite unidentified parameters [30]. It performs calculations by dividing the geometry either in 1D, 2D or 3D domains into subdivisions that are known as mesh elements. These mesh elements remain separated. The nodes that are allotted points at the vertices of elements exist in mesh elements and at these points dependent functions are well-defined. Such nodes provide a more accurate solution for unidentified parameters. The methodology offers efficient strategies to study the system of complex geometries.

### 2.1. Material Design

To study plasmons, first we designed a single GaN nanowire and then shone a light on it. The designed nanowires were 10,000 nm long and 80 nm in diameter. Then following steps were performed to design a model of the nanowire. First, we designed the geometry of the nanowire. A cylindrical shape was used to design the nanowire. After designing the geometry, we added the material gallium nitride to the nanowire. Then, basic properties and some specific properties of GaN were added. Then, physics was applied by choosing the semiconductor module. From the model properties we selected Fermi–Dirac statistics. It is well known that the selection of a study is important, so according to applied physics an appropriate study was selected. For applied semiconductor physics, the study ‘Semiconductor Initialization’ was chosen. This study provides the solution of variables involved in equations. After applying appropriate physics, a study mesh was created which was used for the convergence of studies or to check whether the solution depends on mesh or not. Tetrahedral mesh size was selected. Then, the file was computed after creating the mesh that yielded the design of the GaN nanowire.

### 2.2. Formation of Plasmons

Next, we designed the source of light by using the coefficient PDE module. The light was allowed to shine on the GaN nanowire to form plasmons. For the light source, the input parameters wavelength ‘I’, wave number ‘k’ and flux density were selected. To synthesize the plasmons at the single nanowire, the following procedure was applied. The parameter ‘u’ was used to find absorption. As the geometry of the nanowire was already designed, to shine the light on the nanowire we chose a source of light in the form of a block having a width of 40 nm, depth of 100 nm and height of 100 nm. The material parameters were taken as a relative permittivity of 8.9, thermal conductivity of 30 W/(m·K), density of 6070 kg/m^3^ and heat capacity of 490 J/(kg·K). A union was applied on both the geometries, cylinder and block, after which both geometries were connected to prepare a single geometry.

In order to address the generation and propagation of electric and magnetic fields, COMSOL implements Maxwell’s equations. Upon shining the electromagnetic radiations on surfaces and interfaces, the collective valance electron oscillations to produce the plasmons are generated if specific conditions are met. The dielectric constant considerations are satisfied for the semiconductor/metal interfaces. The propagation of plasmons can be understood via propagation wave vector $k_{P}$ that can be understood via the following relations:

$$k_{p}^{2}+k_{z}^{2}=k^{2}$$

kz2=(ωc)2 (∈m∈d∈m+∈d)

k2=(ωc)2 ∈d

kp2=(ωc)2 (∈d2∈m+∈d)

The constants carry the usual meanings, where $\in_{d}$ and $\in_{m}\;$ are the values of the relative permittivity of the dielectric (semiconductor here) and metal, respectively.

Among different available modules of COMSOL, the Semiconductor and Coefficient Form PDE helps design GaN nanowire to study the propagation of plasmons. The Semiconductor module works using drift diffusion equations under isothermal or non-isothermal models.

For the semiconductor physics for the GaN nanowire physics, the ‘Coefficient Form PDE module’ was added only to the source of light. Our source of light had a block shape and its one side was attached to the nanowire to incident the light. The light travels in the form of a flux in such a way that flux source 1 and flux source 2 were used for input and output. We selected the ‘stationary study’ for the PDE module and the ‘semiconductor initialization’ study for the Semiconductor module. Then, we built the mesh of the entire geometry in such a way that the size of the mesh was chosen by selecting the tetrahedral size. Following the same procedure, we designed more nanowires to study coupled and uncoupled plasmon propagation.

The preparation of the structures and setup was followed by computation of the results. The desired results were obtained from the 3D plot group, which exhibited plasmons in the form of flux density in the geometry. The 1D and 3D plots groups were used to obtain the results.

## 3. Results

The computed results on the formation of plasmons in GaN nanowires upon interaction of light with the semiconductor are described in the following sections.

### 3.1. Single Nanowire

**Figure 1:**
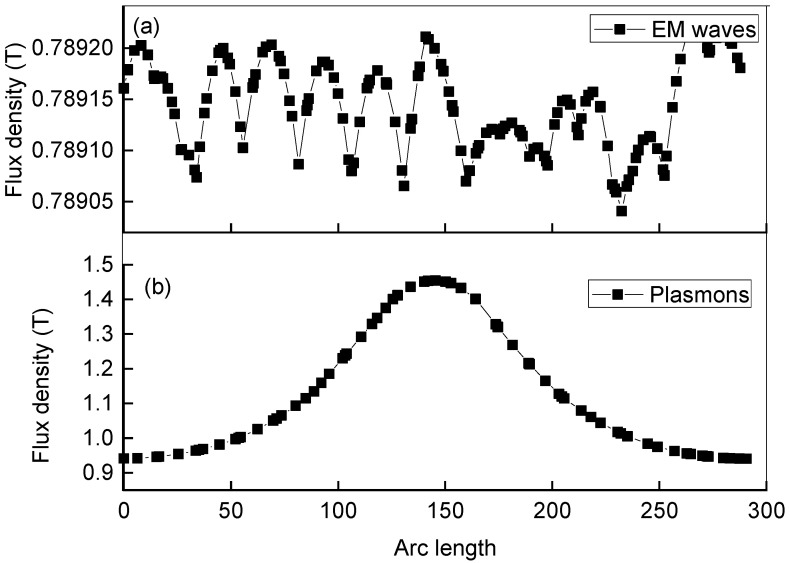
The computed flux density in case of 340 nm wavelength incident light as a function of arc length for EM waves and plasmons. (**a**) Flux density across geometry for incident light (**b**) Flux density in region of plasmons formation.

The collective oscillations comprising plasmons are formed at the interface of the semiconductor nanowire and dielectric medium [30]. To study the formation of the plasmons at the interface, we examined the waveform and flux density of incident electromagnetic waves and plasmons. The EM waves prior to incidence on the structure are shown in Figure 1a, which merely reveals the presence of oscillations. However, upon incidence of the waves on GaN, the flux density recorded at all positions in the geometry is shown in Figure 1b, which points to the presence of a well-defined peak.

The variation in the flux density across the geometry for incident light can be observed, which points to plasmon formation. The plot given in Figure 1b shows the flux density of plasmons in the region of plasmon formation for the case of 340 nm. The flux density of incident EM waves varies in the geometry at different positions and its maximum value is 0.7 T. It appears that, at all fluxes, the density positions are not uniform throughout. Hence, in the case of plasmons, the flux density increased up to 1.4 T, which confirms the formation of plasmons in response to the collective oscillations [30]. The peak at the center of the geometry, i.e., about arc length 150 flux, indicates that the flux density is maximum. The gradual decrease in flux density on both sides of the peak shows the propagation of the plasmons towards the outer boundaries. Hence, the variation of the flux density with arc length reveals the existence of plasmons in the designed geometry and also provides information about the propagation of plasmons [31].

The radiations with different wavelengths in the region 360 nm to 700 nm were allowed to interact with the GaN nanowire to produce light–matter interactions to form plasmons. The colored 3D plots (not shown here), obtained in the form of circular variations, were closely inspected to study plasmon formation. The circular boundaries represent the periphery of the cylindrical nanowire. The color distributions were studied, in light of the color legend, in the region of plasmons which shows different values of the flux density. The yellow color on the boundaries represents the flux density 1.25 T, whereas the red color represents the flux density 1.3 T. As we move further towards the center, we can see the dark red color representing the flux density 1.4 T. This means the flux density is less at the boundaries of the nanowire as compared to the flux density at the center of the nanowire. In the other region of the block, the blue color represents the flux density 1 T.

**Figure 2:**
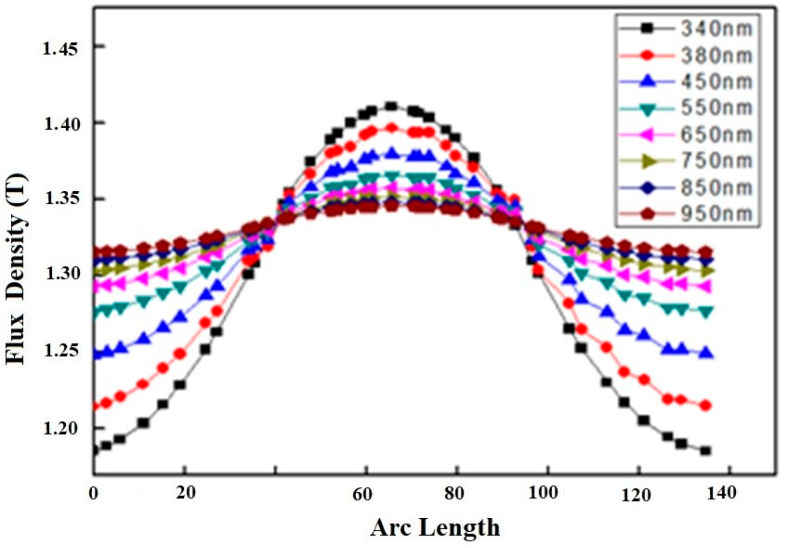
Flux density versus arc length showing propagation of plasmons at different wavelengths from 340 nm to 950 nm. The curves indicate variation of flux density as per the legend. The region 40–100 points to the nanowire placed with source whereas the regions 0–40 and 100–140 indicate boundaries of block.

The propagation of surface plasmons (SPs) in the designed geometry was obtained at all wavelengths in the form of flux density, as shown in Figure 2. The arc length shows the entire region where plasmons propagate after formation. The flux density gradually increases at the start, after which it increases rapidly in the segment from the 40–100 region, which is the center of the geometry where the center of the plasmons exists. Afterwards, a peak is found which represents the maximum flux density and formation of the plasmons. Beyond this region, flux density again varies gradually, indicating the propagation of the plasmons. The peak can be detected for all wavelengths of light, but its intensity is maximum for the case of 340 nm as represented by the black curve.

The slowest variation in flux density can be observed for the wavelength of 950 nm. When wavelength increases, the energy of photons decreases, which points to the incidence of more photons, and hence flux, to the nanowire. This is the reason that at the start the flux density increases with the wavelength as per the observation of the region arc length 0–40. The prepared geometry consists of one block as the source and a cylindrical nanowire, whereas the boundary of the nanowire is connected at the center of the block. When light from one side of the block falls on the nanowire, the interaction of electrons with light produces plasmons. Which are subsequently propagated down the geometry. The block and cylindrical nanowire have specific areas with the block area greater than that of the cylinder. The plasmons that are at the center of the block occupy less area compared to the plasmons near to the walls of the block due to the geometry considerations. The region between 0–40 and 100–140 arc length shows the boundaries of the block, whereas the region 40–100 arc length shows the center of the block where the nanowire interfaces. Due to the area effect, we found a greater flux density at the center shown by the peaks as compared to the flux density present at the sides of block. The peaks become less intense with the increase in wavelength, which indicates that there is a decrease in the flux density with an increase in the wavelength. The flux density becomes nearly equal in all regions, with an increase in the wavelength, because flux density is the ratio of the flux and area. Hence, the optimization of the incident wavelength is required to obtain the same flux density.

### 3.2. Two Nanowires

Above we discuss the propagation of plasmons at the surface of the single GaN nanowire. Similarly, the formation and propagation of plasmons is studied by using two nanowires. The computed results of the coupled and uncoupled nanowire dimmers are discussed below.

(a) Uncoupled Nanowires

**Figure 3:**
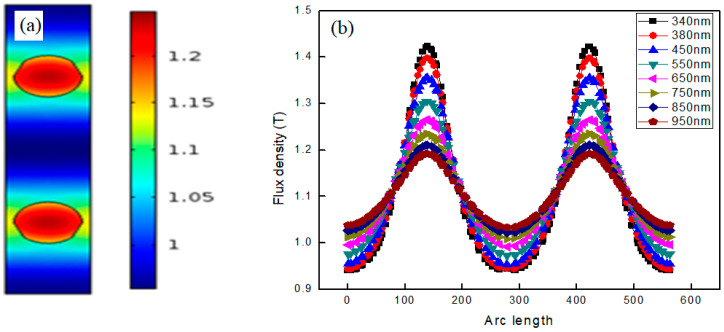
(**a**) Plasmon formation at interfaces of two GaN Nanowires placed at distance of 284 nm by shining light of 340 nm. The color bar represents flux density in region of plasmons. (**b**) Flux density versus arc length showing the existence of plasmons by variation of flux density in the region where plasmons formed at different wavelengths when two nanowires are decoupled and placed at distance of 284 nm.

In order to study the uncoupled structure, both the nanowires were placed 284 nm away from each other to ensure that the boundaries of both nanowires were not in direct contact or interaction. When the 340 nm light shines on the dimmer, the surface plasmons are formed at the interfaces of the nanowires, as shown in Figure 3a. The circles represent the boundary of the nanowire to view the plasmons. The color legend placed on the right side gives the flux density associated with each color and different colors in the form of circles shows the variation of the flux density. The dark red colored patch in the center indicates the flux density above 1.2 T for both plasmons, whereas the green color represents the flux density of about 1.1 T.

As per previous discussions on the single nanowire, the flux density is greater in the center of the nanowire compared to the periphery. It can be said that the plasmons in the case of the uncoupled dimmer start propagation from the high flux density central region to the lower flux density outer region [32]. The only difference is the number of nanowires in the geometry; hence, the finding indicates that the flux density of an uncoupled plasmon is the same as that for the single nanowire.

There is a definite reason behind placing the nanowires at a specific distance of 284 nm. When we moved the nanowires further away from each other than 284 nm, we obtained two plasmons of the same flux density at all further separations. However, when we brought the nanowires closer by reducing the distance, the results were different. As we reduced the distance between nanowires, the plasmonic structure began to change, which points to the mutual interaction of the plasmons. The interaction of the plasmons with each other appeared to be strongly dependent on the separation of the nanowires. The plasmons appearing due to the nanowires were completely decoupled when the distance between the nanowires approached 284 nm. To study the propagation of the plasmons across the region, we plotted flux density versus arc length as shown in Figure 3b.

In the case of uncoupled GaN nanowires, maximum flux density is obtained placed at 340 nm as represented by the black curve in Figure 3b. Two peaks are obtained at the interfaces of both nanowires across the designed geometry where plasmons are formed. The plasmonic flux density is maximum at the center of the nanowire compared to the other positions in the geometry. When the wavelength increases to 380 nm, the maximum flux density is obtained. The peak is obtained at the same location, but its value is reduced when the incident wavelength is reduced to 340 nm. As we further increase the wavelength on the same position, the value of the maximum flux density consistently decreases. The initial value of the flux density near to the boundaries of surface at the 340 nm wavelength is smaller in comparison to that at 380 nm. This points to an increase in the flux density near the boundaries of the nanowire, but the peak value of the flux density at the center of the nanowire decreases with the increase in EM wavelength. This behavior occurs for both plasmons formed at the interfaces of GaN nanowires that are placed at a distance of 284 nm.

It can be clearly seen in Figure 3b, as the wavelength increases from 340 nm to 380 nm, the flux density increases; the starting portion of all curves represent this behavior. The peak of the curve appears higher for low wavelengths, which can be seen by comparing the black color curve (for 340 nm), which has a higher intensity, with red color curve (for 380 nm). Similarly, with a further increase in the wavelength up to 950 nm, the peaks of the curves are found at lower positions. The peak represents the region in the center of the nanowire from which plasmons are propagated down the geometry, and hence the two peaks point to the formation of plasmons in the nanowires. It is therefore found that flux density increases with wavelength and the highest flux density is obtained in the interface region of the nanowire. The results are similar to those of the single nanowire with the only difference being that two peaks corresponding to the plasmons are detected in the case of the uncoupled GaN nanowires dimmer.

(b) Coupled nanowires

**Figure 4:**
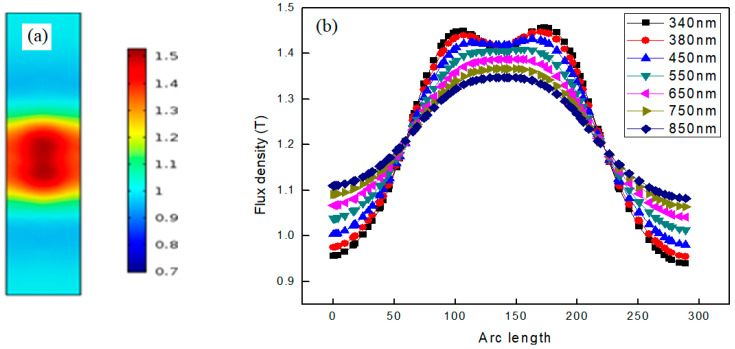
(**a**) Plasmon dimmer when two GaN nanowires were placed closely to show coupling of the plasmons. The color bar shows the flux density at different position in the region. (**b**) The flux density versus arc length for light incident at different wavelengths in case of uncoupled nanowires.

The coupled case is studied in order to understand the interaction of plasmons in the nanowires. The interaction can change the flux density in the geometry and the propagation of plasmons across the formation region can also be different in the case of coupling. When two nanowires are closely placed in the configuration, it ensures the interaction of the plasmons; the computed results are shown in Figure 4a. The resultant plasmon is clearly a combination of two plasmons, which is a consequence of the coupled nanowire dimmer. The interaction causes the linear superposition of the flux density of both plasmons, which produces a single resultant plasmon of higher flux density. When we moved the nanowires away from each other, the flux density of the resultant plasmon gradually decreased. Upon increasing the inter-nanowire separation to 284 nm, two separate plasmons appeared as a result of decoupling the nanowires. Hence, instead of a single highest flux density, an equal flux density was observed at two different positions in the geometry.

In the case of coupling, we have maximum flux density in the center where a dimmer is formed from the superposition of both plasmons. Figure 4b explains the variation in flux density for the coupled plasmons that shows the maximum value at the point where coupling occurs and reduced values at the rest of the positions. As we moved away from the center of the geometry, flux density decreased, which points to the propagation of SPs. From this we can say that plasmons start to propagate from the center and move symmetrically on both sides towards the outer boundary of the geometry. Hence, plasmons are formed at the interfaces of both semiconductor nanowires and propagate towards the outer region with more flux density at the interfaces. At the 340 nm wavelength, the black curve indicates two peaks in the geometry as discussed above; the first peak is related to the plasmon due to the first nanowire whereas the second corresponds to the other nanowire. However, the peaks are still connected, which points to the interaction of the nanowires. For the cases of the 380 nm and 450 nm wavelength, the peaks follow the similar trend of gradually merging. For the case when the wavelength increases to 550 nm, the grey colored curve indicates the complete merging of two peaks and the appearance of a single peak similar to the case of the single nanowire. It seems in reality there are two plasmons formed at the interfaces of two nanowires, but they are totally coupled, due to which we obtain only one peak in that region. For all cases after the 550 nm wavelength, only one resultant peak is obtained, which points to the coupling. It can thus be concluded that, when two nanowires are placed closely together, the plasmons formed by shining a light of 340 nm are interacting and coupled with each other. The formation of a coupled plasmon points to the propagation and interaction of the plasmons formed at the interface of the nanowires dimmer. It can further be said that, upon appearance of a single coupled plasmon, both the plasmons lose their individuality upon the coupling.

The analysis of flux density with changes in arc length for different incident EM waves indicates that the peak of all curves represents the center of the nanowires dimmer. Hence, in the case of the single nanowire and for two uncoupled nanowires, the difference is that in the coupling case plasmons are formed in the dimmer with more flux density, which indicates more photons and ultimately more collective oscillations.

### 3.3. Four Nanowires

In order to further elaborate the coupling of plasmons in the assembly of GaN nanowires, we studied the configuration of four nanowires in the same geometry for the coupled and uncoupled cases.

(a) Uncoupled Nanowires

The assembly of four GaN nanowires with the same parameters was placed in the geometry. First, we placed all the nanowires at a distance to study the uncoupled plasmons at different wavelengths. Then, we placed all nanowires close to each other to study the coupled and interacting plasmons.

**Figure 5:**
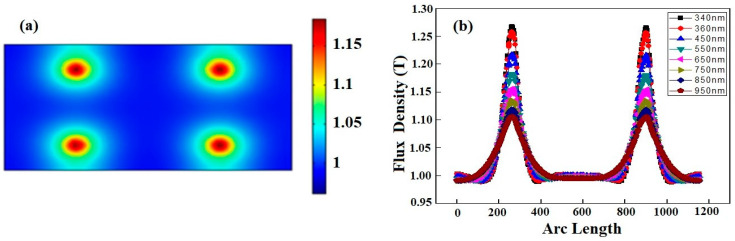
(**a**) Plasmons formed by shining the 340 nm light of on four GaN nanowires placed at some distance. The color bar showing flux density at different positions is given. (**b**) Flux density as a function of arc length showing plasmon formation by shining 340 nm on four GaN nanowires.

The distance between any two of the four nanowires was 250 nm, which was optimized in order to get completely uncoupled plasmons. The light of 340 nm wavelength was allowed to shine on the nanowires to obtain the plasmons formed separately at the interfaces, as shown in Figure 5a. The dark red color at the center of each plasmon shows the maximum flux density, which starts propagation from the center of the nanowire and moves towards the boundaries. From the color legend we can see that for the dark red color the flux density is maximum at the center. This also shows four plasmons propagating in the formation region in the same manner, starting propagation from the center and moving towards the boundaries on the nanowires. The plasmons propagation along the geometry can be seen clearly from the flux density versus arc length curves, which show the flux density at all positions in the geometry given in Figure 5b. At the 340 nm wavelength, a black curve with two peaks was obtained instead of four peaks, as in the case of two nanowires where we also obtained two peaks. It seems here that one peak shows the two plasmons and the second peak shows the other two plasmons. As the wavelength increases the peaks become flat. The two curves are separated, which means there is no interaction between the plasmons.

(b) Coupled Nanowires

**Figure 6:**
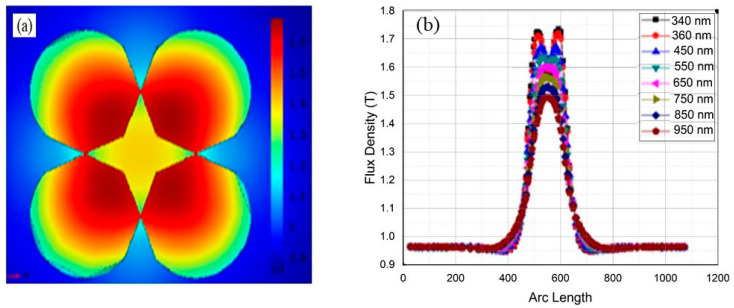
(**a**) The coupled plasmons formed by incidence of light on four GaN nanowires. The color legend shows the flux density at different positions. (**b**) The changes in flux density across geometry where plasmons are formed and propagate at different wavelengths from 340 nm to 950 nm when four GaN nanowires are placed together to study the coupling effects.

In order to study the coupling of four nanowires, we reduced the distance between them to place them in close proximity and shone the light. The plasmons are formed as in the previously mentioned dimmer, which means now they are interacting with each other, as shown in Figure 6a. The same geometry is used here to study the interaction between the plasmons. The dark brown color indicates a greater flux density and association of the plasmons with each other. Hence, the interaction between plasmons triggers when the distance between nanowires is reduced. The complete coupling is observed when four nanowires are placed side by side, whereas the flux density is greater and it is maximum at the center of the nanowire. The plasmons propagate from the center to the boundaries, where they have a greater flux density.

When the wavelength of light changes the plasmons formed with the same shape and also at the same position, only the flux density is changed. Even the propagation of plasmons also remains the same; they start to propagate from the center to the boundaries. The propagation of plasmons is independent of the wavelength, and hence, by changing the wavelength of the incident light, the number of photons in plasmons can be varied. Figure 6b shows the propagation of plasmons across the formation region at all wavelengths. In cases where nanowires are placed in contact with each other, at the 340 nm wavelength two peaks are present but connected with each other, shown by the black color curve in the figure. Hence, the plasmons are not totally coupled; the existence of two peaks points to the presence of more than one plasmon. The same behavior is obtained at the 380 nm, 480 nm and 550 nm wavelength, but as we further increase the wavelength up to 650 nm, one curve with one peak is obtained as shown by the green color curve. It is noted that as the wavelength increases from 340 nm, the two obtained peaks are the same as observed in the low wavelengths. However, there is a small distance between them which is further reduced with an increase in the wavelength until 650 nm, where it vanishes. The mentioned distance shows the presence of four plasmons without any separation, which points to plasmon interaction.

In general, flux density changes in proportion to the wavelength, but in the current case, the flux at the center of nanowire decreases with the increase in wavelength. This behavior is observed for both cases where nanowires are placed in contact or at a certain distance. It is therefore found that the flux density in the case of the four coupled nanowires is greater than that of the uncoupled nanowires. The flux density increases with the wavelength at all positions in the formation region except the nanowire region, where it decreases. It should be noted that, in order to obtain totally coupled plasmons, the incident wavelength plays an important role. In the case of two nanowires the specific wavelength is 550 nm, but in the case of four nanowires it is 650 nm. Hence, the wavelength at which plasmons completely interact with each other (i.e., are completely merged) increases with the increase in the number of interacting nanowires. This points to the possibility of tuning the plasmonics by using a suitable number of coupled nanowires in assembly.

### 3.4. Effect of Coupling and Number of Nanowires

**Figure 7:**
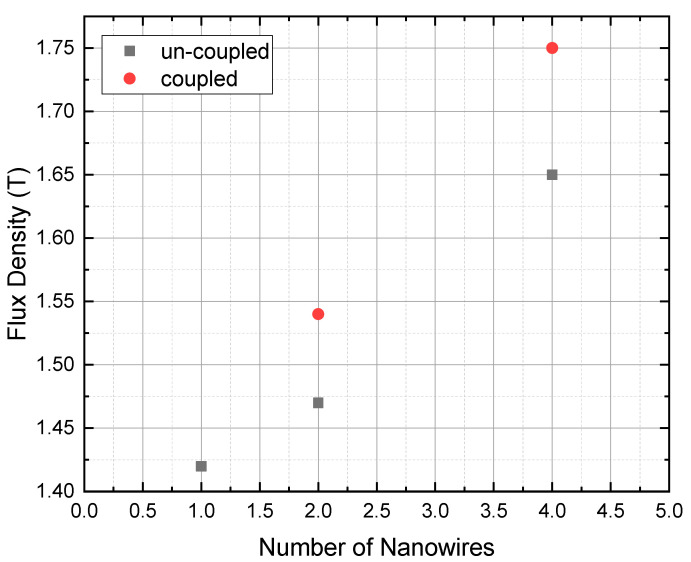
The computed values of flux density for 1, 2 and 4 GaN nanowires. The square blocks represent flux density for uncoupled nanowires and circles represent flux density for the coupled nanowires exhibiting the effects of coupling on flux density with increase in number of nanowires.

The findings pointed out that the number of nanowires affects the coupling and that the flux density increases with the increase in the number of nanowires [33]. In order to explore the relation between the changes in the flux density and number of nanowires we plotted the data as shown in Figure 7. The flux densities obtained for single, double and four nanowires, including the coupled plasmons of two and four nanowires, are helpful to conclude the study. The wavelength of 340 nm being shone to produce single plasmons exhibited the lowest flux density. In the case of two nanowires placed at a distance of 284 nm for the uncoupled case and then placed side by side for the coupled case, with the same incident light, the coupled case exhibited higher flux density. The incidence of the same light on four nanowires exhibited the highest flux density for the coupled assembly in comparison to all the studied cases.

The number of electrons is expected to increase with the increase in the number of nanowires. More electrons absorb more photons of incident light; hence, there will be more collective oscillations [34]. Hence, it can be said that with an increase in the number of nanowires, more collective oscillations and hence a higher flux density are obtained. Thus, to obtain the desired plasmons we can optimize the assembly by the proper choice of semiconductor and the number of nanowires. In the coupled case of two nanowires, shining the light of 360 nm exhibited a flux of 1.52 T, whereas the uncoupled case revealed a flux of 1.46 T. Similarly, in the case of four nanowires the flux density decreased from 1.67 T to 1.74 T. It is also noted that the vertical distance between the second block and first circle is less compared to the third block and second circle in the graph. This means that for two coupled and uncoupled nanowires, there is no large difference between the flux densities of plasmons compared to the case of four nanowires.

The metallic nanoparticles are superior in surface plasmonics when compared with semiconducting nanostructures. However, considering the future devices and applications, the usage of hybrid materials and the integration of different properties in the same chip are needed. Recently, graphene has been predicted for use for applications in photoelectric detection and chemical sensing [35]. Similarly, a design of a tunable absorber based on a semi-metallic metamaterial for bio-chemical sensing has been reported [36]. The study on GaN nanowires and similar materials for which properties are tunable and highly surface dependent are of interest for device grade applications.

## 4. Summary and Conclusions

A detailed theoretical study was conducted to study the formation of plasmons and their coupling in GaN nanowires. It was found that flux density increases with the wavelength and the existence of a high flux density in the region of the nanowire, but the same decreases with the increase in wavelength. The difference in comparison to that of single nanowire is that here two plasmons are present. The flux density of individual plasmons is also the same as that of single nanowire plasmons. The comparison of one and two uncoupled nanowires indicates that in the coupling case the plasmons formed a dimmer with more flux density, which indicates more photons to induce more collective oscillations. The interaction of plasmons with each other appeared strongly dependent on the separation of the nanowires. The number of nanowires affect the coupling effect and we found that flux density increases with an increase in the number of nanowires. It can be said that the wavelength at which plasmons are completely merged increases with an increase in the number of interacting nanowires. The flux density of plasmons can be achieved by optimizing the wavelength of input light, the number of nanowires and the coupling of nanowires. The findings of this study are helpful for the realization of GaN-based plasmonic devices.

## Data Availability

Data sharing is not applicable to this article.
